# The relationship between prevalence and duration of weight loss strategies and weight loss among overweight managed care organization members enrolled in a weight loss trial

**DOI:** 10.1186/1479-5868-3-3

**Published:** 2006-02-17

**Authors:** Jennifer A Linde, Darin J Erickson, Robert W Jeffery, Nicolaas P Pronk, Raymond G Boyle

**Affiliations:** 1 Division of Epidemiology and Community Health, School of Public Health, University of Minnesota, Minneapolis, MN, USA; 2 HealthPartners Research Foundation, Minneapolis, MN, USA

## Abstract

**Background:**

Many adults in the United States report engaging in weight loss behaviors. The current study examined weight loss strategies among managed care organization members, to determine the prevalence and impact of weight loss behaviors in this population. We hypothesized that greater engagement in weight loss strategies would be associated with greater weight loss success.

**Methods:**

Data were taken from Weigh-to-Be (WTB), a two-year weight loss trial (*N *= 1801, 72% female, mean age = 50.7 years, mean weight = 95.9 kg, mean BMI = 34.2 kg/m^2^). Every six months, participants completed a questionnaire assessing frequency and duration of weight loss strategies (calorie reduction, fat reduction, increased fruit/vegetable intake, increased exercise, elimination of sweets, consumption of less food). General linear models and structural equation methods were used to examine associations between weight loss strategy use and weight change over time.

**Results:**

Weight loss strategy prevalence rates ranged from 68% to 76% over two years. For all dietary strategies, any use of the strategy between baseline and 24 months was associated with weight loss at 24 months; those who did not engage in the strategy showed weight gains during that period. Results of general linear models and structural equation models indicated that increased use of weight loss strategies was significantly associated with greater 24-month weight loss.

**Conclusion:**

The prevalence of weight loss strategies in this obese adult managed care population was quite high, and use of these strategies was associated in dose-response fashion with better weight loss. Future interventions may benefit from emphasis on persistence of similar strategies to achieve more successful outcomes.

## Background

Obesity rates in the United States continue to climb, such that two-thirds of adults are classified as either overweight [body mass index (BMI)>25 kg/m^2^] or obese (BMI>30 kg/m^2^) [1]. Recent prevalence estimates from a large-scale national telephone survey (*N *= 184,450) indicate that many U.S. adults are trying to lose weight, with nearly one-half of women (46%) and one-third of men (33%) reporting current weight loss attempts [2].

There is consensus among weight loss experts that adults who attempt weight loss should engage in calorie reduction (500–1000 kilocalorie deficit per day), reductions in fat intake (to less than 30% of total energy intake), and increased physical activity (at least 150 minutes per week at moderate intensity levels) in order to lose weight safely and appropriately [3]. National data indicate that most adults seeking to lose weight appear to engage in recommended activities; in the general population survey noted above, 56% of women and 53% of men reporting calorie reductions, and 66% of women and 69% of men reporting increases in physical activity during weight loss attempts [2].

In another examination of weight loss behaviors of U.S. adults, the Pound of Prevention (POP) study assessed specific weight loss practices of over 1100 community-dwelling women and men (mean age = 35.0 years; mean BMI = 27.2 kg/m^2^) enrolled in a 3-year weight gain prevention trial. At least two-thirds of participants reported increasing exercise (82%), decreasing fat intake (79%), reducing the amount of food eaten (78%), eliminating sweets or "junk food" (73%), reducing calories (73%), or increasing fruit and vegetable intake (67%) in order to control weight. For those reporting engagement in these dietary strategies, greater duration of strategy use was associated with better weight loss over time [4].

The aim of the present study was to examine the prevalence of the specific weight loss strategies assessed in the POP study, in a sample of adults from a managed care population who were actively seeking to lose weight. This study serves to broaden the findings of the POP study by examining weight loss behaviors in an older, heavier sample drawn from the health care system. We hypothesized that, as in the general community, greater engagement in these strategies in this particular adult population would be associated with weight loss success.

## Methods

### Procedure

Data were taken from the Weigh-to-Be project, a collaboration between the University of Minnesota and HealthPartners, a large Minnesota managed care organization (MCO), designed to evaluate phone and mail-based weight loss interventions. The University of Minnesota and HealthPartners Institutional Review Board committees approved the study protocol. The sample was comprised of 508 men and 1293 women who provided baseline data and were enrolled in the study. Participants were randomized to one of three groups: a mail-based weight intervention, a phone-based weight intervention, or a usual care group. Weight loss protocols were designed as potentially cost-effective delivery modes for weight loss within a large health plan population. The intervention modules were offered continuously over the 24 months of the study. After randomization, intervention participants were asked to contact study personnel to activate their assigned module (phone or mail). Following activation, participants spent an average of 5.3 months on phone course activities and an average of 8.8 months on mail-based activities. Participants in the usual care group were not offered weight intervention beyond health promotion programs in the health plan. Details of the study are described elsewhere [5,6].

### Measures

Age, gender, educational attainment, ethnicity, marital status, and smoking status were measured by self-report questionnaire at baseline. During the baseline visit, trained research staff measured height using a wall-mounted stadiometer. Baseline and 24-month weights were measured using a calibrated digital scale. Self-reported weights were collected from participant questionnaires at 6, 12, and 18 months, and were adjusted by +1.5 kg for men and +1.7 kg for women to account for self-report bias [7]. Weight change in kilograms was assessed at 6, 12, 18, and 24 months, and body mass index (kg/m^2^) was computed.

At 6, 12, 18, and 24 months, participants were asked the following item to assess engagement in weight loss strategies: "Indicate whether you did this during the past six months: reduce number of calories eaten, increase exercise levels, increase fruits and vegetables, decrease fat intake, cut out sweets and junk food, reduce amount of food eaten." Duration was assessed by asking participants: "For those items you did in the past six months, also write in the total number of weeks that you did it."

### Data analysis

Statistical analyses were conducted using Statistical Analysis System Version 8.2 (SAS) [8] and Mplus Version 3.12 [9]. Frequencies, indicating engagement in a given behavior during any of the four time intervals (between baseline and 6 months, 6–12 months, 12–18 months, and 18–24 months), were calculated to determine prevalence of weight loss strategy use. Mean durations of strategy engagement (in weeks) were summed across the four intervals, for a possible total of 104 weeks. SAS general linear models were used to examine associations of strategy endorsement or duration with 24-month weight change; models controlled for baseline weight (in kilograms). To assist in interpretation of the significance of these findings, effect sizes are reported in tables using Cohen's *d *statistic (small = .20, medium = .50, large = .80) [10].

Structural equation methodology (SEM) was used to examine the temporal relationship between weight loss strategy use and weight change. SEM has four distinct advantages over the general linear model in this analysis: 1) a latent variable is used to measure the use of weight loss strategies, 2) a model-testing approach directly compares alternative models, 3) temporal effects of the intervention are modeled and compared, and 4) all cases, including those with missing data, can be included in the model if data meet conditions for either missing at random (MAR) or missing completely at random (MCAR). A latent variable representing weight loss strategies was estimated for each of the four time points following baseline based on six manifest indicators. Each manifest indicator was the number of weeks during the previous 6-month period in which individuals employed each of the specific weight loss strategies noted above.

After establishing the fit of the measurement model, a set of nested structural models were estimated and compared. All structural models included a set of baseline exogenous covariates and predictors that were allowed to covary. All post-baseline weight loss strategy latent variables and weight outcomes were regressed on the full set of covariates and predictors. The first structural model included autoregressive paths of lag 1 for both the weight loss strategy latent variables and weight. The next structural model added cross-lagged paths from the weight loss strategy latent variable for a 6-month interval and weight at the end of that interval. The next structural model replaced the cross-lagged paths from weight loss strategy to weight with cross-lagged paths from weight to weight loss strategy for the subsequent 6-month period. The final structural model included both sets of cross-lagged paths. Two relative fit indices, the Akaike Information Criterion (AIC) and the sample-size adjusted Bayesian Information Criterion (SBIC) were used to determine the best-fitting model. Once a final structural model was retained, further constraints were used to compare the magnitude of specific structural paths [11,12].

Missing data were primarily due to attrition. Examination of missing data found a relationship between missingness and a number of variables, including age, marital status, education, ethnicity, and weight at baseline. In order to justify the use of maximum likelihood for missing data in the current analysis, the covariates shown to be associated with missingness were included in the structural model. All weight loss strategy latent variables and weight following baseline were regressed on all covariates, except weight at baseline, which was associated only with weight at 6 months.

## Results

### Sample demographics

The study population had a mean age of 50.7 (12.4) years. The majority (91%) was white, most participants (78%) had completed some college education, and 70% were married. Nearly one-half of participants (47%) had ever smoked, and 9% reported currently smoking. Eighty-six percent had ever dieted and over two-thirds were clinically obese (mean baseline BMI = 34.2 kg/m^2^, mean baseline weight = 95.9 kg). Mean weight changes at 6, 12, and 18 months were -0.92 (5.18) kg, -1.27 (6.10) kg, and -1.58 (7.12) kg, respectively. By the 24-month study follow-up, participants had lost 1.35 (7.22) kg on average.

By 24 months, 56% of the initial 1801 participants (*N *= 1000) remained in the study. As compared to those who were present at baseline only, those also present at 24 month follow-up were older [mean age = 52 vs. 49 years, *t*(1684) = -6.90, *p *< .0001], weighed less [mean BMI = 33.8 vs. 34.7 kg/m^2^, *t*(1671) = 3.38, *p *< .001], were more likely to be married [73 vs. 67%, χ^2^(1) = 8.41, *p *< .01], white [93 vs. 88%, χ^2^(1) = 14.35, *p *< .001] and college-educated [80 vs. 74%, χ^2^(1) = 9.13, *p *< .01]. Adjusting for these variables in general linear models (presented in Tables 2 and 3 below) did not affect the magnitude or statistical significance of findings.

**Table 1:** Prevalence and duration of weight loss strategies over two years in the study sample.

Strategy	Prevalence (total *N *for strategy)	Mean Duration in Weeks (*SD*)
Reduce calories	76.4% (of 1347)	27.2 (20.9)
Increase exercise	70.4% (of 1243)	24.7 (19.1)
Increase fruits/vegetables	74.3% (of 1296)	31.4 (22.7)
Decrease fat intake	74.9% (of 1309)	31.2 (24.0)
Cut sweets/junk food	67.9% (of 1193)	26.0 (21.1)
Reduce amount of food	77.4% (of 1360)	27.6 (21.4)

*Note*. Means are indicated for those who reported engaging in strategies. For all strategies, range = 1–104 weeks.

**Table 2:** Endorsement of weight loss strategies and 24-month weight change.

Strategy Endorsement (*N *= 1000)*		*n*	Adjusted Mean (kg)	*SE*	*t*	*p*	*d*†
Reduce calories:	Yes	882	-1.68	0.23	4.49	<.0001	.28
	No	118	1.58	0.69			
Increase exercise:	Yes	828	-1.46	0.24	1.16	.25	.08
	No	172	-0.75	0.57			
Increase fruits/vegetables:	Yes	864	-1.60	0.24	3.13	.002	.20
	No	136	0.59	0.66			
Decrease fat intake:	Yes	853	-1.67	0.24	3.78	.0002	.24
	No	147	0.87	0.63			
Cut sweets/junk food:	Yes	778	-1.83	0.24	4.27	<.0001	.26
	No	222	0.52	0.49			
Reduce amount of food:	Yes	881	-1.68	0.23	4.42	<.0001	.28
	No	119	1.59	0.70			

*Note*. Strategies were entered into separate general linear models. All analyses controlled for baseline weight (in kg).

*N = 1000 due to missing values.

†Cohen's *d *= effect size statistic (small = .20, medium = .50, large = .80).

**Table 3:** Associations between the duration of weight loss strategies and 24-month weight loss.

Strategy (*N *= 778–882)*	*B*	*SE (B)*	*t*	*p*	*d*†
Reduce calories	-0.11	0.01	-10.32	<.0001	.70
Increase exercise	-0.10	0.01	-8.52	<.0001	.58
Increase fruits/vegetables	-0.06	0.01	-5.94	<.0001	.41
Decrease fat intake	-0.06	0.01	-6.26	<.0001	.43
Cut sweet/junk food	-0.08	0.01	-7.33	<.0001	.54
Reduce amount of food	-0.10	0.01	-9.45	<.0001	.63

*Note*. Strategies were entered into separate general linear models. All analyses controlled for baseline weight (in kg).

*N = 778–882 due to missing values.

†Cohen's *d *= effect size statistic (small = .20, medium = .50, large = .80).

Prevalence and duration of weight loss strategies in this sample are reported in Table 1. The majority of participants reported engaging in these strategies over the assessed 2-year period, and for 23–30% of the time (24–31 weeks out of 104).

### Association of weight loss behaviors and weight change over time

#### General linear models

Associations of weight loss strategy endorsement with 24-month weight change are presented in Table 2. For all dietary strategies, any use of the strategy between baseline and 24 months was associated with weight loss at 24 months, and those who did not engage in each strategy showed weight gains during that period. Effect sizes were small (*d *= .20–.28) but statistically significant. The only exception was for increased exercise, which did not reach statistical significance. Examination of adjusted means shows that those who did not increase exercise during the 24 months lost some weight, although not as much as those who reported increasing exercise during the study period.

Associations of weight loss strategy duration with 24-month weight change are presented in Table 3. In all cases, results were statistically significant, with effect sizes in the small to medium range (*d *= .41–.70). Models indicated that one-week increases in engagement in weight loss strategies were associated with small decreases in weight, up to 0.11 kg, which has the potential to translate to a weight loss of 3–6 kg over the course of a year.

#### Structural equation models

**Figure 1:**
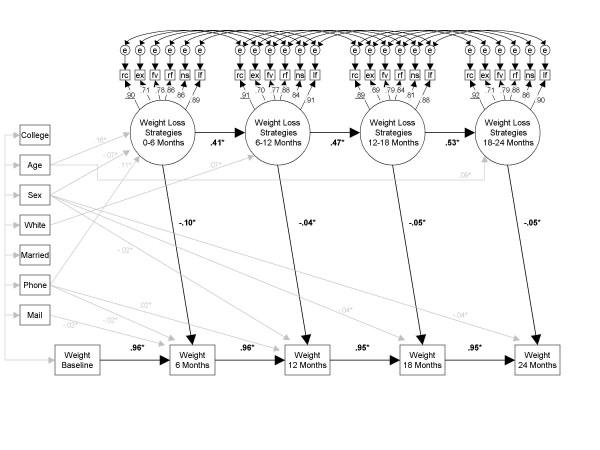
**Structural equation model of weight loss behaviors and weight change**. *Note*. *N *= 1801. rc = reduce calories, ex = increase exercise, fv = increase fruit and vegetable intake, rf = reduce fat intake, ns = cut out sweets and junk food, lf = reduce amount of food eaten. Error covariances are represented in the figure by the letter e. An asterisk (*) denotes statistical significance, *p *< .05.

Four nested structural models were compared. All models included covariances between all weight loss strategy latent variables, 'striped' error covariances (covariances between errors associated with like indicators for bounded time periods), and constrained factor loadings for like indicators over the four time points after baseline (providing factorial invariance over time). After considering the relative magnitude of both AIC and SBIC, Model 2 was retained as the best-fitting structural model (*df *= 508, loglikelihood = -88591.68, AIC = 178083.01, SBIC = 178532.93). Figure 1 shows a graphical representation of this final structural model. This model included autoregressives of lag length 1 for both weight loss strategy and weight, and cross-lagged paths from weight loss strategy use over a 6-month period to weight at the end of that 6-month period. Figure 1 includes only those paths from covariates that are statistically significantly larger than zero, although all paths were modeled. The factor loadings were all statistically significant and of acceptable magnitude, with standardized loadings ranging from 0.69 for increased exercise to 0.92 for calorie reduction. The majority of error covariances were statistically significant and positive, with correlations ranging from 0.01 to 0.16.

Autoregressive paths ranged from 0.41 to 0.53 for weight loss strategies and 0.95 to 0.96 for weight. Controlling for the strong autoregressive effect of weight, increased use of weight loss strategies was significantly associated with reductions in weight. Standardized effects ranged from a high of -0.10 for the first 6-month interval to a low of -0.04 for the second 6-month interval. Using additional model constraints, follow-up analyses showed that the magnitude of the effect during the first 6-month interval was statistically significantly larger than the 3 subsequent intervals, which themselves do not differ.

## Discussion

As obesity rates increase in the U.S. there is a pressing need to identify healthy behavioral strategies that not only are successful for controlling weight, but that individuals are willing and able to adopt. The present study examined a potential set of useful strategies (calorie reduction, fat reduction, increased fruit/vegetable intake, increased exercise, elimination of sweets, consumption of less food) in a population of overweight and obese adults seeking weight loss in a managed care setting.

Prevalence of any engagement in the target weight loss strategies in this sample was quite high, and the type and frequency of strategy use are comparable with observations of weight loss strategy use in more general community samples [2,4]. This behavior engagement suggests that those who seek to lose weight act, for the most part, in a manner that is consistent with the behaviors recommended by experts as safe and appropriate for weight loss [3]. Use of strategies demonstrated a dose-response association with 24-month weight change. The observation of greater weight change at 24 months appears to validate self-reports of strategy use, in that intended outcomes (i.e., weight loss) were achieved more readily by those who reported having used these strategies. Results of structural equation models that used maximum likelihood estimation to account for missing data due to substantial attrition (44%) in this sample replicated those from general linear models, thus bolstering the validity of the findings.

In this dataset, approximately one out of every four days in a given time period was spent engaging in at least one weight loss strategy, which is comparable to data on weight loss strategy use in an adult community sample [4]. In the context of development and implementation of future weight loss intervention efforts, it may be important to consider devising interventions that emphasize greater persistence in applying similar strategies to those reported here, in order to achieve more successful outcomes.

The present data indicated that all strategies were fairly popular, with prevalence rates in a narrow range from 67.9% for elimination of sweets and junk food to 77.4% for reducing the amount of food eaten. However, effect sizes for dose-response associations between strategy use and weight change varied somewhat, with small effects for two strategies (decreasing fat intake and increasing fruit/vegetable intake) and medium effects for the remaining four strategies (reducing calories or amount of food eaten, increasing exercise, and eliminating sweets/junk food), suggesting that some strategies may be more efficacious than others. Future studies may consider exploring further the differential likelihood of strategy adoption, persistence, or relative efficacy over the course of a weight loss attempt.

A notable limitation of this study is the 44% attrition rate from baseline to 24 months. However, adjustment for variables that were predictive of attrition (i.e., age, baseline BMI, marital status, ethnicity, educational attainment) did not alter the findings of either general linear models or structural equation models. In addition, the structural equation methodology employed maximum likelihood techniques to account further for missing data. Therefore, we are confident that our findings are a reasonable representation of overweight members of the managed care population here who are interested in enrolling in an MCO-offered weight loss program.

## Conclusion

The prevalence of engaging in some form of weight loss strategy over a 24-month period in this managed care population is high. However, individuals appear to spend only about 1 day in 4 engaged in weight loss. These data are closely comparable with those on weight loss strategies in an adult community sample [4] and are similar to national data in this domain [2]. Importantly, reported use of weight loss strategies demonstrated a dose-response association with measured weight change. Future research directions might include the development of interventions that emphasize persistence of similar strategies to achieve more successful outcomes, either in similar populations or in other groups of interest, such as older or younger adults and adolescents, or those seeking weight loss from sources other than managed care settings, such as community centers, other public or private organizations, or by self-direction.

## Competing interests

The author(s) declare that they have no competing interests.

## Authors' contributions

JL performed statistical analyses and drafted the manuscript. DE participated in development of statistical models, performed statistical analyses, and drafted sections of the manuscript. RJ, NP, and RB conceived of the study and participated in its design and coordination and reviewed manuscript drafts. All authors read and approved the final manuscript.
